# Suicide stigma and suicide literacy among Bangladeshi young adults: a cross-sectional study

**DOI:** 10.3389/fpsyt.2023.1160955

**Published:** 2023-05-12

**Authors:** Ishrat Jahan, Azaz Bin Sharif, A. B. M. Nahid Hasan

**Affiliations:** Department of Public Health, North South University, Dhaka, Bangladesh

**Keywords:** suicide stigma, suicide literacy, young adults, suicide, adults

## Abstract

**Introduction:**

Suicide is one of the leading causes of death worldwide. Owing to poor suicide literacy, people are not aware of the consequences of the suicide stigma, which may affect individuals. This study aimed to examine the status of suicide stigma and literacy among young adults in Bangladesh.

**Methods:**

This cross-sectional study included 616 male subjects and female subjects residing in Bangladesh aged between 18 and 35 years who were invited to complete an online survey. Suicide literacy and suicide stigma among the respondents were assessed by using the validated Literacy of Suicide Scale and Stigma of Suicide Scale, respectively. Other independent variables that have been found to affect suicide stigma or literacy were included in this study based on prior research. Correlation analysis was used to assess the relationships between the study's main quantitative variables. Multiple linear regression models were used to assess factors affecting suicide stigma and suicide literacy, respectively, after controlling for covariates.

**Results:**

The mean literacy score was 3.86. The participants' mean scores in the stigma, isolation, and glorification subscale were 25.15, 14.48, and 9.04, respectively. Suicide literacy was negatively associated with stigmatizing attitudes (*p* = 0.005). Male subjects, unmarried/divorced/widowed, less educated (below HSC), smokers, less exposure to suicide, and respondents with chronic mental illness had lower suicide literacy and more stigmatizing attitudes.

**Conclusion:**

The findings suggest that addressing suicide literacy and stigma by developing and executing awareness programs on suicide and mental health among young adults may increase knowledge, decrease stigma, and hence prevent suicide among this population.

## Introduction

Suicide is one of the leading causes of death worldwide. There are an estimated 800,000 cases of suicide deaths per year throughout the world (1). In addition, suicide attempts are many folds higher than the actual number of suicides (1). Suicide not only affects the people who attempted it but also leaves a long-term scar on the people surrounding them. Suicide is a serious yet preventable public health problem if proper awareness, help-seeking behavior, and counseling regarding mental health problems can be outreached (2).

Over 79% of all suicides occur in low- and middle-income countries (LMICs) (1). The World Health Organization (WHO) estimates that ~20% of global suicides are caused by pesticide poisoning, with the majority occurring in the rural areas of LMICs (1). Mental disorders are perceived to be one of the significant causes of suicide in developed nations. But, in the LMIC crisis, moments such as interpersonal relationship problems are considered to be major causes of suicide (1, 3, 4). Most of the LMICs did not create any national suicide prevention strategies or programs (1). Bangladesh, a densely populated country in Southeast Asia, has achieved health-related Millennium Development Goals (MDG), but suicide is still under-addressed (5).

According to the WHO, among all age groups, young adults are more susceptible to suicides, and it is ranked the second most cause of death among adults aged between 15 and 29 years (1). Another study reported that in young adults actual suicide attempts have become more common and are key indicators of further suicidal risk (6). A scoping review in Pakistan conducted by Shekhani et al. (7) reported that suicidal behaviors were more common among individuals younger than 30 years. A systematic review conducted in Bangladesh revealed that the mortality rate of suicide was 39.6 per 100,000 among all age groups, and the commonly affected age group was 20–29 years (8).

Multiple factors are responsible for suicidal behaviors, such as a higher level of stigma toward suicidal behavior, inadequate suicide or mental health-related literacy, and cultural representation (9–12). The term “stigma” is used to describe a “mark of disgrace; stain, as on one's reputation” (13). Suicide stigma is regarded as one of the barriers to asking for psychological assistance and making suicide prevention efforts (14, 15). In Bangladesh, there is a significant cultural and religious taboo around suicide, and individuals who attempt or die by suicide are often stigmatized and ostracized by their communities (5, 16). This can lead to a lack of social support for those who are struggling with suicidal thoughts and may discourage individuals from seeking help or disclosing their suicidal ideation to others (17). By addressing suicide stigma, individuals and communities can create a more supportive and accepting environment for those who are struggling with suicidal thoughts, reducing the risk of social isolation and increasing the likelihood of help-seeking behavior (18).

Additionally, improving suicide literacy can help individuals and communities to better identify and respond to suicide risk (11). Suicide literacy has been defined by understanding the four components of suicidality: warning signs and symptoms, causes, risk factors and treatment, and preventive methods (11). Suicide literacy is inadequate among the young generation, which has not been widely explored (19). Improving suicide literacy may enable people and communities to identify individuals who may be at risk for suicide based on their behavior, emotions, and other risk factors (20). This can lead to early intervention and support, which can help prevent suicidal behavior (21). A cross-sectional study conducted by Ludwig et al. (22) among the German adult population revealed a moderate level of suicide literacy, low level of stigmatization, low normalization of suicide, and lower attribution of suicide to isolation. Another study among Arab young adults observed high suicide stigma, low suicide literacy, and negative attitude toward psychological help-seeking (23). A higher level of suicide stigma and suicide literacy was found among the Australian adult farming community in a study performed in 2018 (24). A study conducted by Shah et al. (25) revealed better suicide literacy scores among Nepali physicians and nurses. In Bangladesh, there is a lack of awareness about suicide risk factors and limited knowledge of effective suicide prevention strategies and resources (26). A study conducted by Arafat et al. (27) among Bangladeshi university students on validation of the Stigma of Suicide Scale-Short Form (SOSS-SF) and Literacy of Suicide Scale-Short Form (LOSS-SF) revealed a low literacy score about suicidality and a high level of stigma. Another study conducted by Maruf et al. (28) among Bangladeshi doctors reported higher suicide literacy among single doctors with a family history of suicide and a history of suicidal thoughts in their lifetime, while suicide stigma was lower among the respondents with a history of mental illness. Evidence elaborates that with the rise in seeking professional help from psychiatrists, there is a decline in stigma and an increase in literacy about mental health that ultimately lowers the number of suicidal cases (29).

Although research on suicide has grown in recent years in Bangladesh, there is a lack of studies that investigated the status and associated factors of suicide stigma and literacy among young adults (5, 27, 28, 30–33). The study conducted by Arafat et al. (27) included only university students and focused on the validation of the SOSS-SF and LOSS-SF scales. There is another study on suicide stigma and suicide literacy among physicians in Bangladesh that assessed the factors associated with suicide stigma and suicide literacy among that specific study population (28). Yet no study looked upon the factors associated with suicide stigma and suicide literacy among young adults. This study included students as well as other professionals within the age range of 18–35 years. By focusing on this population, the study provides important insights into the factors that may contribute to suicide risk among this group. Therefore, this study aimed to assess the magnitude of suicide stigma and suicide literacy among Bangladeshi young adults and the factors associated with it.

The ecological systems theory (EST) serves as the conceptual framework for the present study (34). Individuals are at the center of the paradigm, with different tiers of systems layered around them (35). The concept splits a person's environment into five major systems/levels (36). These levels are interconnected and can be evaluated jointly (37, 38), with each level impacting the other, and its influence depends on its relationship with other levels during the life development of an individual (37). For instance, an individual's knowledge, beliefs, and attitudes surrounding mental health concerns (e.g., suicide) may be influenced by others and the cultural and religious aspects of his or her environment. Using this model, this study assessed the sociodemographic characteristics, suicide literacy levels, and suicide stigma levels, in addition to their relationships, among a sample of Bangladeshi young adults.

## Methods

### Study participants and recruitment procedure

A cross-sectional study was carried out among young adults in Bangladesh between January 2022 and April 2022. Participants aged between 18 and 35 years living in Bangladesh were included in the study. Those who refused to respond or did not confront to inclusion criteria were excluded from the study. Young adults have been defined as the age group of 18–35 years in this study (39–42). A convenient sampling technique was used to recruit participants. To mitigate possible biases, a large sample was recruited, and the questionnaire was circulated using social media sites (Facebook and Messenger) to reach a diverse segment of the community. The sample size was calculated using the formula of Cochran's ($(n=\frac{z^{2}\;*p\;(1-p)}{e^{2}}).$ With a 5% of margin of error (*e*), estimated population proportion of 61.7% (*p*) (27), and standard normal deviation of 1.96 (*z*), the required sample size was 362. But the study team tried to reach a large sample than the required sample size.

A Google Form questionnaire was developed to ascertain the status of suicide stigma and literacy among young adults. A data collector from each division (eight divisions were taken to represent the young population of Bangladesh) circulated the link through different social media sites. The authors considered that individuals who live in different divisions might have many friends and relatives living in the same division, and the likelihood of receiving a response from them would be higher. The survey included a direct link to the online Google Form and a brief description of the study's objective, purpose, and eligibility criteria. Data collectors reached participants by creating a post on Facebook describing the study and providing the link to the survey. They also shared the survey on Messenger by directly messaging individuals who meet the inclusion criteria. Participants provided consent by clicking “yes” to agree to participate in the survey. Participation was anonymous, with no financial incentive offered. To avoid the repetition of responses from the same individual, the authors limited the Google form to a single response so that responders could respond once. Finally, 636 questionnaires were completed with self-reported online responses. After cleaning the dataset and discarding the missing responses, a dataset of 616 responses was analyzed for this study. The ethical approval committee, the Institutional Review Board (IRB) of North South University (NSU), approved the study on August 2022 (#2022/OR-NSU/IRB/0807) against the second author as principal investigator.

### Measures

Participants completed a questionnaire comprising socio-demographic data, personal experience with suicidality, and validated versions of the 16-item SOSS-SF and the 12-item LOSS_SF. The SOSS-SF and LOSS-SF scales have previously been validated in a university-setting sample in Bangladesh (27). This study only used validated questionnaires similar to Arafat et al. for quantifying the suicide literacy and stigma assessments. In addition, the authors have considered other explanatory variables associated with suicide literacy and stigma. Specifically, this study examines the relationship between suicide literacy, stigma, and other variables, such as chronic physical illness, psychological help received, and smoking or substance history among individuals. The questionnaire was initially composed in English and later translated into Bengali by a language expert. In all, 5% of the study's total sample was used for pilot testing of the English questionnaire before translating it to Bengali.

### Sociodemographic profile

The sociodemographic section of the questionnaire obtained information regarding respondents' age, gender, religion, the highest level of education, occupation, and marital status. The age by which usually students in Bangladesh complete graduation is 22–23 years. Therefore, the authors tried to compare individuals who were ≤23 and individuals who were above 23. In addition, information on smoking status and any substance use history were collected.

### Personal experience with suicidality and other diseases

The second section comprised questions related to respondents' personal experience with suicidality and other diseases, such as the history of chronic physical or mental illness, relationship with parents or spouse, family history of suicide or suicidal attempt, personal history of suicidal thought or attempt, and history of taking any psychological help.

### Stigma of suicide scale-short form

SOSS-SF was employed to measure the respondent's stigma toward suicidal people based on the original instrument with 58 items and a 16-item reduced version (10). In accordance with the original scale, there were three subscales of this scale: “stigma,” “isolation/depression,” and “glorification/normalization.” The SOSS-SF scale consists of sixteen different descriptors; each item consists of a one-word descriptor of a person who dies by suicide, such as “strong,” “lonely,” and “immoral,” all of which were assessed on a 5-point Likert scale (strongly disagree, disagree, neutral, agree, and strongly agree). These categories were coded from 1 to 5 ranging from strongly disagree to strongly agree, respectively. Summing up the item scores, the total SOSS score was calculated with a possible range of values from 16 to 80. Among the three subscales, the stigma subscale consists of eight items with scores ranging from 8 to 40, the isolation subscale consists of four items with scores ranging from 4 to 20, and the glorification subscale consists of four items with scores ranging from 4 to 20. There are no cutoff values for the subscales of SOSS-SF. A higher score on the SOSS-SF scale indicates a greater stigma toward suicide.

### Literacy of suicide scale-short form

The LOSS-SF scale was used to assess the respondents' literacy level toward suicide a 12-item instrument, based on the original instrument with 26 items (43). The LOSS-SF includes four knowledge categories: (a) reasons or nature of suicide (four items), (b) risk factors for suicidal conduct (three items), (c) symptoms and signs (three items), and (d) treatment and prevention (two items). This scale consists of suicidality-related items with three possible answers (true, false, and I don't know). Alcoholism was substituted with substance dependence in item number 4 of LOSS-SF. LOSS-SF was linguistically updated as studies conducted in Bangladesh have revealed that other types of drugs are considered substantial risk factors for suicide (33). The scoring system of item 11 on the LOSS-SF scale was reversed in this study because studies revealed that women are more at risk of suicide than men in Bangladesh (8, 31). In the LOSS-SF employed in the present study, items 2, 4, 6, and 8 include true assertions, whereas the remaining items are false. To ensure consistency with the original instrument, correct responses were assigned a score of 1; incorrect or “I don't know” responses were assigned a score of 0 in LOSS-SF in this study. Total LOSS scores (ranges from 0 to 12) were determined by adding the item scores of each individual. There are no cutoff values for the LOSS-SF scale. Those who scored more were considered to have better literacy toward suicide.

### Statistical analysis

The sociodemographic characteristics and suicidal attitude-related variables were demonstrated with frequency and percentages. Correct responses of the LOSS-SF and agreement statements of SOSS-SF were expressed in frequencies. The normality of the data was tested by the Shapiro–Wilk test, and the data were normally distributed. Pearson's correlation was performed to observe the association between the LOSS-SF score and three subscales of the SOSS-SF score. Independent-samples *t*-test or one-way ANOVA was used for continuous variables when comparing means of two or greater than two groups. Finally, multiple linear regression models were used to assess factors affecting suicide stigma and suicide literacy after controlling for covariates. The independent variables which were described as predictors of suicide stigma and literacy in previous studies were included in this study for regression analysis. All tests were two-tailed, and a *p*-value ≤0.05 were considered statistically significant. The internal consistency of the SOSS-SF score was checked by Cronbach's alpha coefficient. Due to the response pattern (yes, no, and do not know), the internal consistency form of reliability was not justified for LOSS-SF. The SOSS-SF instrument had an internal consistency of 73% during the pilot study. Statistical software IBM SPSS Statistics (RRID: SCR_019096) was used for all statistical analyses.

## Results

Among 616 participants, the majority of the participants were male (56.2%), Muslim (88%), and single (73.4%). The percentage of respondents belonging to age groups 18–23 years and 24–35 years were 40.9%, and 59.1%, respectively. The respondents had an average age of 24.5 years (SD = 3.39 years). The majority of the participants were studying at an undergraduate level or above (64.4%). More than half of the participants (51.9%) were students, 23.4% were service holders, and the rest (24.7%) were from other professions. The larger portion of the study population was non-smoker (82.1%) and had no history of alcohol/other substance use (90.3%) (Table 1).

**Table 1:** Socio-demographic profile of the study participants (*N* = 616).

**Variable**	**Categories**	***n*** **(%)**
Age (years)	18–23	252 (40.9)
	24–35	364 (59.1)
Gender	Male	346 (56.2)
	Female	270 (43.8)
Religion	Muslim	542 (88.0)
	Others	74 (12.0)
Education level	Up to primary	52 (8.4)
	SSC	16 (2.6)
	HSC	151 (24.5)
	Bachelor and above	397 (64.4)
Employment status	Student	320 (51.9)
	Service holder	144 (23.4)
	Others	152 (24.7)
Marital status	Married	161 (26.1)
	Single (unmarried/divorced/widowed)	455 (73.9)
Smoking status	Smoker	110 (17.9)
	Non-smoker	506 (82.1)
Alcohol or substance use history	Yes	60 (9.7)
	No	556 (90.3)

SSC, Secondary School Certificate; HSC, Higher Secondary School Certificate.

Suicide related experience and other clinical factors are presented in Table 2. The vast majority of individuals did not suffer from any form of persistent physical ailment (89.4%) or mental illness (92.0%). Among the surveyed individuals, 97.2 and 93.8% reported having healthy relationships with their parents and spouse or children, respectively. No prior suicidal ideation or suicide attempts were observed among the majority of the participants (83.6%), whereas the rest had a history of suicidal thoughts or attempts (16.4%). With regard to seeking psychological help, only a few (8.0%) respondents reported having sought psychological support in the past. The percentage of the participants having a family history of suicide or suicidal attempts and a family history of mental illnesses were 10.2 and 19.1%, respectively.

**Table 2:** Suicide-related experience and other clinical factors (*N* = 616).

**Variable**	**Categories**	***n*** **(%)**
Chronic physical condition	Yes	65 (10.6)
	No	551(89.4)
Chronic mental illness	Yes	49 (8.0)
	No	567 (92.0)
Relationship with parents	Good	599 (97.2)
	Bad	17 (2.8)
Relationship with spouse/children	Good	152 (93.8)
	Bad	10 (6.2)
History of suicidal thought/attempt	Yes	101 (16.4)
	No	515 (83.6)
History of taking any psychological help	Yes	49 (8.0)
	No	567 (92.0)
Family history of suicide/suicidal attempt	Yes	63 (10.2)
	No	553 (89.8)
Family history of mental illness	Yes	117 (19.0)
	No	499 (81.0)

The agreement percentage of the items of the stigma of suicide scale is presented in Supplementary Table S1. Overall, the instrument had a strong internal consistency (Cronbach's alpha of 0.703), with the stigmatization subscale having an alpha of 0.81, the normalization/glorification subscale having an alpha of 0.60, and the isolation/depression subscale having an alpha of 0.74. Three of the eight items in the stigma subscale were endorsed by more than 50% of the sample, while the remaining five items were endorsed by more than 30% of the sample, with the exception of one item (vengeful), which was endorsed by ~20% of the respondents. The mean stigmatization subscale score was 25.15 ± 6.16, indicating that the participants stigmatized suicidal individuals. The participants' mean isolation subscale score was 14.48 ± 2.91, indicating that they strongly agreed that isolation is associated with suicide. Over 70% of the respondents endorsed two items relating to social isolation. Two additional items were endorsed by more than 40% of the sample. The participants' mean score on the glorification subscale was 9.04 ± 2.69, indicating they did not believe suicidal individuals should be praised or normalized. Three items of the glorification subscale were supported by 4%−10% of the participants, while the remaining item (brave) was agreed by more than 28% of the sample.

**Figure 1:**
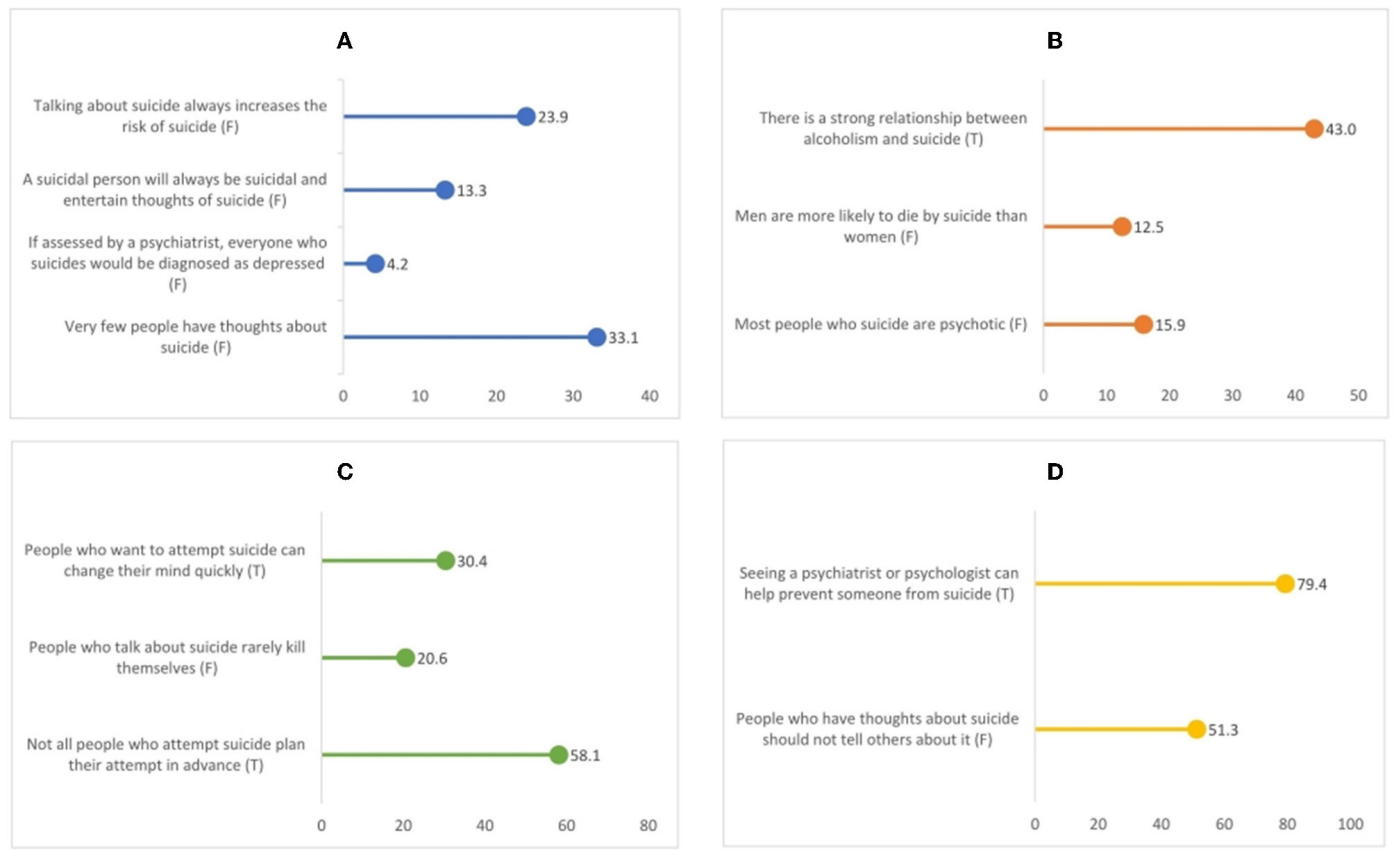
Literacy of Suicide Scale-SF items with four domains ordered by percent correct. **(A)** Causes/nature. **(B)** Risk factors. **(C)** Signs. **(D)** Treatment/prevention. SF, short form; T, Correct response is True; F, correct response is False.

Figure 1 illustrates the findings of the study population for all LOSS-SF items categorized by the knowledge domain. The overall sample had a mean LOSS-SF score of 3.86 (SD = 1.94). The majority of the participants were observed to have a very poor suicide literacy score. More than 50 and 70% of the participants provided correct responses to two items linked to the domain “treatment and prevention.” On the other hand, fewer than 5% of the respondents selected the statement “if assessed by a psychiatrist, everyone who suicides would be diagnosed as depressed” as false. The rest of the items did not have a percentage of accurate responses over 50%, except the “not all people who attempt suicide plan their attempt in advance” item in the knowledge domain, “signs” (58%).

The correlation assessment in the Supplementary Table S2 reveals that literacy of suicide was negatively associated with stigmatizing attitudes (*p* = 0.005) and normalizing or glorifying suicidal ideation, while it was positively associated with attribution to depression and isolation. Within SOSS-SF, the stigmatizing attitude was found to be positively associated with the isolation subscale (*p* < 0.001) and negatively associated with the glorification subscale (*p* < 0.001). The isolation subscale was negatively associated with the glorification subscale (*p* = 0.001).

A bivariate analysis of LOSS-SF and subscales of SOSS-SF by sociodemographic characteristics, behavioral factors, and suicide-related factors are shown in Tables 3, 4. Significant differences in the mean LOSS score between groups were observed for gender (p = 0.005), education (p = 0.019), smoking status (p < 0.001), and history of suicidal thoughts/attempts (p < 0.001). Significant differences in the mean stigmatization score between groups were found for gender (p < 0.001), education (p < 0.001), profession (p < 0.001), substance use (p = 0.007), chronic mental illness (p = 0.001), family history of suicide (p = 0.036), family history of mental illness (p = 0.005), history of suicidal thoughts/attempts (p < 0.001), and history of taking previous psychological help (p = 0.003).

**Table 3:** Comparison of mean LOSS-SF score and SOSS-SF scores by sociodemographic characteristics and behavioral factors (*N* = 616).

**Variable**	***n***	**LOSS-SF**	**SOSS-SF**		
			**Stigma**	**Isolation**	**Glorification**
	**Total**	**Mean (SD)**	**Mean (SD)**	**Mean (SD)**	**Mean (SD)**
**Age (in years)**					
18–23	252	3.7 (2.0)	24.8 (6.3)	**14.8 (3.0)**	8.8 (3.0)
24–35	364	3.9 (1.9)	25.4 (6.0)	**14.3 (2.8)**	9.2 (2.4)
**Sex** ^a^					
Female	270	**4.1 (2.0)**	**24.0 (6.0)**	14.6 (2.7)	9.1 (2.7)
Male	346	**3.7 (1.9)**	**26.0 (6.2)**	14.4 (3.1)	9.0 (2.7)
**Religion** ^a^					
Muslim	541	3.9 (1.9)	25.3 (6.0)	14.5 (2.9)	**8.9 (2.7)**
Others	75	3.6 (1.8)	24.2 (6.9)	14.0 (2.8)	**9.7 (2.4)**
**Education** ^b^					
Up to primary	52	**3.1 (1.0)**	**28.9 (3.0)**	13.6 (1.4)	9.6 (1.5)
SSC	16	**3.5 (1.3)**	**27.4 (5.3)**	14.3 (2.2)	10.4 (3.2)
HSC	151	**3.8 (2.1)**	**24.5 (6.6)**	14.7 (3.2)	9.0 (3.0)
Bachelor and above	397	**3.9 (1.9)**	**24.8 (6.2)**	14.5 (3.0)	8.9 (2.7)
**Employment status** ^b^					
Student	320	3.8 (2.0)	**24.2 (6.2)**	**14.5 (3.2)**	**9.0 (2.9)**
Service holder	144	4.0 (1.9)	**25.5 (6.2)**	**15.0 (2.9)**	**8.7 (2.6)**
Others	152	3.9 (1.9)	**26.8 (5.8)**	**14.0 (2.2)**	**9.6 (2.2)**
**Marital status** ^a^					
Married	161	4.0 (1.8)	25.6 (5.5)	**14.1 (2.5)**	9.1 (2.2)
Others	455	3.8 (2.0)	24.9 (6.4)	**14.6 (3.0)**	9.1 (2.8)
**Smoking status** ^a^					
Smoker	110	**3.3 (1.8)**	25.4 (6.4)	14.0 (3.0)	9.4 (2.6)
Non-smoker	506	**4.0 (1.9)**	25.1 (6.1)	14.6 (2.9)	8.9 (2.7)
**Other substance use** ^a^					
Yes	60	3.7 (2.2)	**23.1 (6.4)**	14.0 (2.9)	**10.0 (2.9)**
No	556	3.9 (1.9)	**25.4 (6.1)**	14.5 (2.9)	**8.9 (2.7)**

Bold value indicates p-value of < 0.05.

LOSS-SF, Literacy of Suicide Scale-Short Form; SOSS-SF, Stigma of Suicide Scale-Short Form; SSC, Secondary School Certificate; HSC, Higher Secondary School Certificate; SD, standard deviation.

a Independent t-test for variables with two different categories.

b One-way ANOVA for variables with more than two categories.

**Table 4:** Comparison of mean LOSS-SF score and SOSS-SF scores by suicide-related factors (*N* = 616).

**Variable**	***n***	**LOSS-SF**	**SOSS-SF**		
			**Stigma**	**Isolation**	**Glorification**
	**Total**	**Mean (SD)**	**Mean (SD)**	**Mean (SD)**	**Mean (SD)**
**Chronic physical condition** ^a^					
Yes	65	4.0 (2.1)	24.8 (6.4)	14.9 (2.9)	9.5 (3.1)
No	551	3.8 (1.9)	25.2 (6.1)	14.4 (2.9)	9.0 (2.6)
**Chronic mental illness** ^a^					
Yes	49	4.0 (2.4)	**22.4 (7.2)**	15.1 (2.9)	**10.5 (3.0)**
No	567	3.8 (1.9)	**25.4 (6.0)**	14.4 (2.9)	**8.9 (2.6)**
**Relationship with parents** ^a^					
Good	599	3.9 (1.9)	25.2 (6.2)	14.5 (2.9)	**8.9 (2.7)**
Bad	17	3.7 (2.4)	24.7 (6.7)	15.1 (2.4)	**10.9 (2.2)**
**Family history of suicide/suicidal attempts** ^a^					
Yes	63	4.0 (2.0)	**23.6 (6.1)**	14.8 (2.8)	9.5 (2.7)
No	553	3.8 (1.9)	**25.3 (6.1)**	14.4 (2.9)	9.0 (2.7)
**Family history of mental illness** ^a^					
Yes	117	4.1 (2.1)	**23.7 (6.3)**	14.6 (2.8)	9.1 (2.6)
No	499	3.8 (1.9)	**25.5 (6.1)**	14.4 (2.9)	9.0 (2.7)
**History of suicidal thoughts/attempts** ^a^					
Yes	101	**4.6 (2.0)**	**21.9 (5.9)**	14.7 (3.0)	**9.8 (2.7)**
No	515	**3.7 (1.9)**	**25.8 (6.0)**	14.4 (2.9)	**8.9 (2.7)**
**Any psychological help taken previously** ^a^					
Yes	49	4.3 (2.2)	**22.6 (6.5)**	14.7 (2.9)	**9.9 (2.5)**
No	567	3.8 (1.9)	**25.4 (6.1)**	14.5 (2.9)	**9.0 (2.7)**

Bold value indicates p-value of < 0.05.

LOSS-SF, Literacy of Suicide Scale-Short Form; SOSS-SF, Stigma of Suicide Scale-Short Form; SD, standard deviation.

a Independent t-test for variables with two different categories.

As can be seen in the regression output in Table 5, more literacy was found among those who completed HSC [β = 0.24, *p* = 0.005, 95% CI: (0.32, 1.84)] or were at their bachelor's or above degree [β = 0.25, *p* = 0.003, 95% CI: (0.34, 1.72)], and having a history of suicidal thought/attempt [β = 0.15, *p* = 0.001, 95% CI: (0.33, 1.25)], while comparatively poor literacy was found among smokers [β = −0.18, *p* = 0.001, 95% CI: (−1.40, −0.39)].

**Table 5:** Suicide literacy, subscales of suicide stigma regressed on sociodemographic factors, behavioral factors, and suicide-related factors (*N* = 616).

	**LOSS-SF**		**Stigmatization**		**Isolation**		**Glorification**	
	β	**95% CI**	β	**95% CI**	β	**95% CI**	β	**95% CI**
Age (24–35 years)	0.06	[−0.18, 0.68]	−0.04	[−1.78, 0.86]	−0.11	[−1.30, 0.01]	0.05	[−0.30, 10.28]
Gender (male)	−0.04	[−0.48, 0.20]	**0.11**	[0.30, 2.40]	0.00	[−0.51, 0.53]	−0.02	[−0.57, 0.37]
Education (SSC)	0.06	[−0.41, 1.80]	−0.01	[−3.61, 3.22]	0.02	[−1.29, 2.09]	0.06	[−0.58, 2.48]
Education (HSC)	**0.24**	[0.32. 1.84]	–**0.17**	[−4.81, −0.13]	0.06	[−0.77, 1.54]	−0.10	[−1.66, 0.43]
Education (bachelor and above)	**0.25**	[0.34, 1.72]	–**0.18**	[−4.39, −0.15]	0.09	[−0.50, 1.59]	−0.13	[−1.67, 0.23]
Religion (Muslim)	0.05	[−0.20, 0.74]	0.04	[−0.70, 2.20]	0.04	[−0.36, 1.07]	–**0.09**	[−1.42, −0.12]
Employment (student)	−0.08	[−0.81, 0.17]	–**0.18**	[−3.76, −0.72]	−0.05	[−1.01, 0.49]	−0.09	[−1.19, 0.17]
Employment (service holder)	−0.05	[−0.71, 0.27]	−0.04	[−2.12, 0.89]	0.10	[−0.07, 1.42]	−0.10	[−1.32, 0.03]
Marital status (others)	−0.06	[0.68, 0.19]	0.08	[−0.21, 2.49]	0.06	[−0.28, 1.05]	0.04	[−0.37, 0.84]
Smoking status (yes)	–**0.18**	[−1.39, −0.39]	0.02	[−1.16, 1.94]	−0.06	[−1.24, 0.29]	−0.04	[−0.10, 0.39]
Alcohol/other substance use (yes)	0.06	[−0.25, 1.01]	–**0.11**	[−4.23, −0.34]	−0.04	[−1.31, 0.62]	0.09	[−0.03, 1.72]
Chronic physical condition (yes)	0.05	[−0.24, 0.81]	0.01	[−1.35, 1.87]	0.08	[−0.03, 1.57]	−0.00	[−0.74, 0.70]
Chronic mental illness (yes)	−0.03	[−0.86, 0.38]	−0.04	[−2.89, 0.96]	0.06	[−0.30, 1.61]	**0.10**	[0.14, 1.86]
Relationship with parents (bad)	−0.03	[−1.30, 0.61]	0.05	[−1.26, 4.64]	0.00	[−1.40, 1.52]	**0.10**	[0.25, 2.89]
Family history of suicide/suicidal attempts (yes)	−0.05	[ −0.85, 0.27]	−0.01	[−2.03, 1.45]	0.03	[−0.63, 1.10]	0.04	[−0.47, 1.09]
Family history of mental illness (yes)	0.02	[−0.32, 0.55]	−0.06	[−2.23, 0.46]	−0.01	[−0.75, 0.58]	−0.06	[−0.98, 0.22]
History of suicidal thoughts/attempts (yes)	**0.15**	[0.33, 1.25]	–**0.16**	[−4.85, −1.22]	0.00	[−0.68, 0.72]	0.07	[−0.10, 1.17]
Any psychological help taken (yes)	0.03	[−0.40, 0.77]	−0.04	[−2.67, 0.94]	0.01	[−0.81, 0.97]	0.06	[−0.19, 1.43]

Bold value indicates p-value of < 0.05.

β, standardized coefficient; CI, confidence Interval; LOSS-SF, Literacy of Suicide Scale-Short Form.

In regard to stigmatizing attitudes, while subjects who were students [β = −0.19, *p* = 0.004, 95% CI: (−3.76, −0.72)], had a history of alcohol or other substance use [β = −0.11, *p* = 0.02, 95% CI: (−4.23, −0.34)], and having a history of suicidal thought/attempt [β = −0.16, *p* < 0.001, 95% CI: (−4.05, −1.22)] were associated with reduced stigma; male respondents [β = 0.11, *p* = 0.01, 95% CI: (0.30, 2.40)] were observed to have more stigmatizing attitudes toward suicide (Table 5).

## Discussion

The current study aimed to investigate the magnitude and distribution of suicide stigma and suicide literacy among Bangladeshi young adults. This study also aimed to examine a range of risk factors having associations with suicide stigma or literacy.

In terms of suicide stigma, the results showed that the respondents had a comparatively higher degree of stigma against suicidal individuals (Supplementary Table S1). This outcome is identical to what Bangladeshi (27) and Jordanian studies among university students (23) found, and it is also similar to what researchers in Turkey (44), China (45), and Qatar (46) observed. In the rural farming community of Australia, it was found that the levels of stigma were higher, attributing suicide to isolation and normalization were lower than in the community sample (24). In contrast, an Australian study found that both the general public and university students had less stigma about suicide (10, 11). The multivariate analysis in the current study found less stigmatization among certain groups of people, including students, alcohol or other substance users, and those with a history of suicidal thoughts or attempts. One possible explanation for these findings is that individuals who had personal experience with suicide or suicidal tendencies might have a greater understanding and empathy toward individuals who experience suicidal thoughts and behaviors. They might also have a greater awareness of the complex factors that contribute to suicide risk and may be less likely to hold stigmatizing attitudes and beliefs. Regarding the finding that students reported lower levels of suicide stigma, it is possible that their exposure to mental health resources and education through multiple platforms (academic and non-academic) may contribute to greater awareness and understanding of suicide and mental health issues among themselves compared with other professionals (47). The finding that alcohol or other substance users reported lower levels of suicide stigma is more complex and may be due to a variety of factors. Substance use and mental health issues often co-occur, and individuals who struggle with substance use disorders may have a greater understanding of the complexities of mental health issues and less stigmatizing attitudes toward individuals who experience suicidal thoughts and behaviors (48).

Overall, this study revealed a very lower score of suicide literacy (3.86) among young adults in Bangladesh (Figure 1). For instance, a study conducted among Bangladeshi university students reported a similar score (4.27) (27), while a German study found better suicide literacy among its population (22). A study conducted among Indian health professionals also revealed a better suicide literacy score (19). Exposure to mental health-related knowledge among health professionals might have a positive impact on suicide literacy. Compared with other studies that used the LOSS-SF among the general population and university students, such as the ones in Australia (10, 11), China (45), Turkey (44), and Jordan (9), the current study detected the lowest literacy level. The study suggests that the young age of the participants may be a factor in their limited knowledge of suicide. Another possible explanation could be that the people in the sample had a lot of negative feelings or stigma about suicidal people, which could have also affected their knowledge about suicide (Supplementary Table S1). In terms of sample characteristics and suicide literacy, respondents with higher education, female respondents, and respondents who reported having previous suicidal thoughts or attempts in their lifetimes performed better on the LOSS-SF (Table 5). The existing pieces of literature on suicide literacy (10, 11) and mental health literacy in general (49, 50) provide support for these findings. Female respondents were found to have a higher level of literacy toward suicide in the current study, similar to the findings in earlier studies on suicide literacy (11, 12, 27, 51). Female respondents are generally more likely to seek help and access mental health resources compared with male respondents (52), which could contribute to their better suicide literacy level. Those with higher education levels tend to have greater access to information and resources, which can increase their understanding of mental health issues, which may help them to have better knowledge about suicide (53). Participants who experienced suicidal thoughts or attempts in the past may have greater personal insight into the warning signs and risk factors for suicide. This increased awareness might help them better understand the questions and concepts presented in the LOSS-SF, leading to better performance. According to the multiple knowledge domains and the characteristics of those surveyed, suicide literacy varied significantly. It was found that knowledge of warning signs increases the likelihood that suicidal individuals would seek treatment (54), recommending that such literacy domain should be prioritized, for instance, by informative resources, training programs for particular groups, or activities aimed at increasing awareness.

According to the results, there is a statistically significant inverse association between suicide literacy and the stigma subscale of the SOSS-SF (Supplementary Table S2). This relationship indicates that individuals with a limited understanding of suicide are more likely to have stigmatizing behaviors toward it. A study carried out on Arab young adults (23) concluded similar findings. Several studies in different countries have shown how important it is to improve literacy to decrease stigmatizing attitudes (10, 11, 44, 55).

Several limitations existed in this study. First, the use of an online survey and voluntary sampling method may have resulted in selection bias, as only young individuals who had access to the internet and the survey link had a chance to participate in this study. Second, the cross-sectional study design provided information about the status and the relationships between the study variables at a particular time point and hence would not facilitate establishing causal inference. Third, as suicide is a sensitive and taboo subject, it was impossible to rule out social desirability bias. Another limitation is that the majority of participants in the current study were found to be students, despite the age range covered in the study not typically found within educational settings. The current study did not inquire about the economic status of the study participants, which is another limitation of this study. Despite these limitations, this study provides valuable information on suicide stigma and suicide literacy among Bangladeshi young adults.

The finding in the current study needs more research to figure out how stigma has evolved and what impact suicide literacy has on attitudes and stigma. Future studies could focus on longitudinal studies to examine the changes in suicide stigma and literacy over time. This could provide insights into the effectiveness of interventions aimed at reducing stigma and increasing literacy. Possible research might also evaluate the efficacy of various educational and awareness programs and materials designed to reduce suicide stigma and increase suicide literacy through intervention studies. This study also implies that efforts combating stigma and raising awareness regarding suicide must focus on young adults. Finding out the appropriate prevention strategy for the country is an immediate necessity to formulate, initiate, implement, and evaluate its effectiveness. Males may need more interactive strategies to help them reduce suicidal stigma. One way to increase knowledge about suicide and mental health resources is to offer suicide gatekeeper training to students and other professionals. Interactive workshops or gatekeeper training where adults could learn how to intervene and practice doing so may help to lessen the negative beliefs surrounding the behavior. To date, gatekeeper training has not been started in Bangladesh, and also, no community mental health services team has been formulated in the country (30). This gatekeeper program improves knowledge about suicide, increases self-efficacy to intervene, and improves attitudes about intervention (56). Raising awareness among the general population through mass media should be considered as a potential prevention strategy. Therefore, a multisectoral approach to raising awareness and developing programs by involving government, institutions, and organizations could have a better impact on improving suicide literacy and reducing suicide stigma.

## Conclusion

This study indicated that among a cohort of young adults, suicide stigma was high, and suicide literacy was extremely low. Higher education, being a student, being alcoholic or other substance users, and having a history of suicidal ideation or attempt were associated with less stigmatizing attitudes and better suicide literacy. Furthermore, suicide literacy was negatively correlated with stigmatizing attitudes; as a result, disseminating knowledge could contribute to reducing suicide stigma in the community. The outcome of this study highlighted the necessity of planning health education programs aiming at increasing suicide literacy, which might increase awareness and decrease stigma among young adults.

## Data availability statement

The raw data supporting the conclusions of this article will be made available by the authors, without undue reservation.

## Ethics statement

This study received ethical approval from the Institutional Review Board (IRB) of North South University (NSU). The patients/participants provided their electronic written informed consent to participate in this study.

## Author contributions

IJ and AH carried out the literature search, outlined the data collection procedure, and led the field implementation of the study and were responsible for data entry. IJ, AS, and AH conceived and designed the study. IJ and AS oversaw its implementation, analysis, write-up, planned the statistical analyses, and verified the underlying data. AH prepared Tables 1, 2. AS prepared Table 3 and Figure 1, reviewed, and edited the first draft of the manuscript. IJ prepared the rest of the tables and wrote the first draft of the manuscript. All authors read and approved the manuscript.

## References

[1] World Health Organization (WHO). Suicide. (2021). Available online at: https://www.who.int/news-room/fact-sheets/detail/suicide (accessed December 18, 2021).

[2] Pedersen ER Paves AP. Comparing perceived public stigma and personal stigma of mental health treatment seeking in a young adult sample. Psychiatry Res. (2014) 219:143. 10.1016/j.psychres.2014.05.017 24889842 PMC4086709

[3] Amitai M Apter A. Social aspects of suicidal behavior and prevention in early life: a review. Int J Environ Res Public Health. (2012) 9:985–94. 10.3390/ijerph9030985 22690178 PMC3367292

[4] Jacob KS. The prevention of suicide in India and the developing world the need for population-based strategies. Crisis. (2008) 29:102–6. 10.1027/0227-5910.29.2.102 18664236

[5] Shah MMA Ahmed S Arafat SMY. Demography and risk factors of suicide in Bangladesh: a six-month paper content analysis. Psychiatry J. (2017) 2017:1–5. 10.1155/2017/3047025 29130035 PMC5654290

[6] Hawton K Witt KG Salisbury TLT Arensman E Gunnell D Hazell P. Psychosocial interventions following self-harm in adults: a systematic review and meta-analysis. Lancet Psychiatry. (2016) 3:740–50. 10.1016/S2215-0366(16)30070-0 27422028

[7] Shekhani SS Perveen S Hashmi DS Akbar K Bachani S Khan MM. Suicide and deliberate self-harm in Pakistan: a scoping review. BMC Psychiatry. (2018) 18:44. 10.1186/s12888-017-1586-6 29433468 PMC5809969

[8] Ferdous MZ Alam ASMM. Present situation of suicide in Bangladesh: a review, *medRxiv [Preprint]*. (2021). 10.1101/2021.02.23.21252279

[9] Aldalaykeh M Dalky H Shahrour G Rababa M. Psychometric properties of two Arabic Suicide Scales: stigma and literacy. Heliyon. (2020) 6:e03877. 10.1016/j.heliyon.2020.e03877 32373752 PMC7193320

[10] Batterham PJ Calear AL Christensen H. The stigma of suicide scale: psychometric properties and correlates of the stigma of suicide. Crisis. (2013) 34:13–21. 10.1027/0227-5910/a000156 22846447

[11] Batterham PJ Calear AL Christensen H. Correlates of suicide stigma and suicide literacy in the community. Suicide Life-Threatening Behav. (2013) 43:406–17. 10.1111/sltb.12026 23556504

[12] Calear AL Batterham PJ Christensen H. Predictors of help-seeking for suicidal ideation in the community: risks and opportunities for public suicide prevention campaigns. Psychiatry Res. (2014) 219:525–30. 10.1016/j.psychres.2014.06.027 25048756

[13] Butler S Delbridge A. The Macquarie dictionary, its history and its editorial practices. Lexikos. (2012) 9:152–71. 10.5788/9-1-920

[14] Niederkrotenthaler T Reidenberg DJ Till B Gould MS. Increasing help-seeking and referrals for individuals at risk for suicide by decreasing stigma: the role of mass media. Am J Prev Med. (2014) 47:S235–43. 10.1016/j.amepre.2014.06.010 25145745

[15] Sudak H Maxim K Carpenter M. Suicide and stigma. Acad Psychiatry. (2008) 32:136–42. 10.1176/appi.ap.32.2.136 18349334

[16] Shahnaz A Bagley C Simkhada P Kadri S. Suicidal behaviour in Bangladesh: a Scoping literature review and a proposed public health prevention model. Open J Soc Sci. (2017) 05:254–82. 10.4236/jss.2017.57016

[17] Masoomi M Hosseinikolbadi S Saeed F Sharifi V Jalali Nadoushan AH Shoib S. Stigma as a barrier to suicide prevention efforts in Iran. Front Public Health. (2023) 10:1026451. 10.3389/fpubh.2022.1026451 36699938 PMC9868841

[18] Calati R Ferrari C Brittner M Oasi O Olié E Carvalho AF. Suicidal thoughts and behaviors and social isolation: a narrative review of the literature. J Affect Disord. (2019) 245:653–67. 10.1016/j.jad.2018.11.022 30445391

[19] Ram D Chandran S Basavana GH. Suicide and depression literacy among healthcare profession students in tertiary care center in South India. Psychiatry Behav Sci. (2017) 7:149. 10.5455/jmood.20170830064910

[20] Stone DM Crosby AE. Suicide prevention. Am J Lifestyle Med. (2014) 8:404–20. 10.1177/1559827614551130 30166972 PMC6112615

[21] World Health Organization?. Preventing Suicide: A Global Imperative. Libr Cat Data. (?2014) 89. Available online at: https://apps.who.int/iris/handle/10665/131056 (accessed 24 March, 2023).

[22] Ludwig J Dreier M Liebherz S Härter M von dem Knesebeck O. Suicide literacy and suicide stigma - results of a population survey from Germany. J Ment Health. (2021) 31:517–23. 10.1080/09638237.2021.1875421 33522335

[23] Al-Shannaq Y Aldalaykeh M. Suicide literacy, suicide stigma, and psychological help seeking attitudes among Arab youth. Curr Psychol. (2021) 2021:1–13. 10.1007/s12144-021-02007-9 34177209 PMC8214717

[24] Kennedy AJ Brumby SA Versace VL Brumby-Rendell T. Online assessment of suicide stigma, literacy and effect in Australia's rural farming community. BMC Public Health. (2018) 18:1–12. 10.1186/s12889-018-5750-9 29980237 PMC6035410

[25] Shah S Neupane D Sah KK. Literacy of suicide among doctors and nurses at a tertiary care hospital in Nepal. J Coll Med Sci. (2022) 18:93–102. 10.3126/jcmsn.v18i2.44471

[26] Yasir Arafat SM. Current challenges of suicide and future directions of management in Bangladesh: a systematic review. Glob Psychiatry. (2018) 2:09–20. 10.2478/gp-2019-0001

[27] Arafat SMY Hussain F Hossain MF Islam MA Menon V. Literacy and stigma of suicide in Bangladesh: scales validation and status assessment among university students. Brain Behav. (2022) 12:e2432. 10.1002/brb3.2432 34856071 PMC8785610

[28] Maruf MM Shormi FR Sajib MWH Acharjee P Ara H Roy S. Level and associated factors of literacy and stigma of suicide among Bangladeshi physicians: a cross-sectional assessment. Ment Illn. (2022) 2022:1–8. 10.1155/2022/9914388

[29] Peel R Buckby B McBain KA. Comparing the effect of stigma on the recognition of suicide risk in others between Australia and Brazil. GSTF J Psychol. (2017) 3:1–10. 10.5176/2345-7872_3.2_43

[30] Arafat SMY Saleem T Edwards TM Ali SA Khan MM. Suicide prevention in Bangladesh: the role of family. Brain Behav. (2022) 12:1–7. 10.1002/brb3.2562 35398979 PMC9120730

[31] Arafat SMY. Females are dying more than males by suicide in Bangladesh. Asian J Psychiatr. (2019) 40:124–5. 10.1016/j.ajp.2018.10.014 30309753

[32] Arafat S. Suicide in Bangladesh: a mini review. J Behav Health. (2017) 6:66. 10.5455/jbh.20160904090206

[33] Arafat SMY Mohit MA Mullick MSI Kabir R Khan MM. Risk factors for suicide in Bangladesh: case–control psychological autopsy study. BJPsych Open. (2021) 7:1–5. 10.1192/bjo.2020.152 33323152 PMC7791560

[34] Ecological systems theory. APA PsycNet. (1992). Available online at: https://psycnet.apa.org/record/2004-22011-010 (accessed 26 March, 2023).

[35] Tanhan A. Acceptance and commitment therapy with ecological systems theory: addressing Muslim mental health issues and wellbeing. J Posit Psychol Wellbeing. (2019) 3:197–219. 10.47602/jpsp.v3i2.172

[36] Bronfenbrenner U Evans GW. Developmental science in the 21st century: emerging questions, theoretical models, research designs and empirical findings. Soc Dev. (2000) 9:115–25. 10.1111/1467-9507.00114

[37] Tanhan A Francisco VT. Muslims and mental health concerns: a social ecological model perspective. J Community Psychol. (2019) 47:964–78. 10.1002/jcop.22166 30730559

[38] Tanhan A Strack RW. Online photovoice to explore and advocate for Muslim biopsychosocial spiritual wellbeing and issues: ecological systems theory and ally development. Curr Psychol. (2020) 39:2010–25. 10.1007/s12144-020-00692-6

[39] Petry NM. A comparison of young, middle-aged, and older adult treatment-seeking pathological gamblers. Gerontologist. (2002) 42:92–9. 10.1093/geront/42.1.92 11815703

[40] Ip IMH Honey A McGrath M. ‘Doing' dating: a cross-sectional survey of young adults (18–35 years) in Australia and Hong Kong. Aust Occup Ther J. (2022) 69:233–42. 10.1111/1440-1630.12785 35040135

[41] Aged 18-35? Tell us why your generation is so blighted | Society | The Guardian. (2015). Available online at: https://www.theguardian.com/society/2015/oct/08/aged-18-35-tell-us-why-your-generation-is-so-blighted (accessed 3 April, 2023).

[42] Young, Adults (Ages 18-35) | UUA.org. Available online at: https://www.uua.org/young-adults (accessed 3 April, 2023).

[43] Calear AL Batterham PJ Trias A Christensen H. The literacy of suicide scale: development, validation, and application. Crisis. (2022) 43:385–90. 10.1027/0227-5910/a000798 34128704

[44] Öztürk A. Evaluation of suicide knowledge level and stigma attitudes towards people who committed suicide in university students. J Psychiatric Nurs. (2018) 9:6–104. 10.14744/phd.2018.49389

[45] Han J Batterham PJ Calear AL Wu Y Shou Y van Spijker BA. Translation and validation of the Chinese versions of the suicidal ideation attributes scale, stigma of suicide scale, and literacy of suicide scale. Death Stud. (2017) 41:173–9. 10.1080/07481187.2016.1214633 27715477

[46] Zolezzi M Bensmail N Zahrah F Khaled SM El-Gaili T. Stigma associated with mental illness: perspectives of university students in Qatar. Neuropsychiatr Dis Treat. (2017) 13:1221–3. 10.2147/NDT.S132075 28533684 PMC5431692

[47] En J Goh ML Fern S. Heliyon Mental health awareness of secondary schools students : mediating roles of knowledge on mental health, knowledge on professional help, and attitude towards mental health. Heliyon. (2023) 9:e14512. 10.1016/j.heliyon.2023.e14512 36950622 PMC10025912

[48] Common Comorbidities with Substance Use Disorders Research Report Part 1 : The Connection Between Substance Use Disorders Mental Illness. (2020). Available online at: https://www.ncbi.nlm.nih.gov/books/NBK571451/ (accessed April 4, 2023).

[49] Hadjimina E Furnham A. Influence of age and gender on mental health literacy of anxiety disorders. Psychiatry Res. (2017) 251:8–13. 10.1016/j.psychres.2017.01.089 28189082

[50] Piper SE Bailey PE Lam LT Kneebone II. Predictors of mental health literacy in older people. Arch Gerontol Geriatr. (2018) 79:52–6. 10.1016/j.archger.2018.07.010 30107312

[51] Oliffe JL Hannan-Leith MN Ogrodniczuk JS Black N Mackenzie CS Lohan M. Men's depression and suicide literacy: a nationally representative Canadian survey. J Ment Health. (2016) 25:520–6. 10.1080/09638237.2016.1177770 27128307

[52] Sagar-Ouriaghli I Godfrey E Bridge L Meade L Brown JSL. Improving mental health service utilization among men: a systematic review and synthesis of behavior change techniques within interventions targeting help-seeking. Am J Mens Health. (2019) 13:1–18. 10.1177/1557988319857009 31184251 PMC6560805

[53] Uddin MN Bhar S Islam FMA. An assessment of awareness of mental health conditions and its association with socio-demographic characteristics: a cross-sectional study in a rural district in Bangladesh. BMC Health Serv Res. (2019) 19:1–11. 10.1186/s12913-019-4385-6 31409332 PMC6692949

[54] Goldney RD Fisher LJ Wilson DH Cheok F. Mental health literacy of those with major depression and suicidal ideation: an impediment to help seeking. Suicide Life-Threat Behav. (2002) 32:394–403. 10.1521/suli.32.4.394.22343 12501964

[55] Abuhammad S Hamaideh S. Nursing students' attitudes toward seeking professional psychological help before and after attending a mental health course. Nurs Educ Perspect. (2022) 43:129–31. 10.1097/01.NEP.0000000000000796 33660683

[56] Aldrich RS Cerel J Drapeau CW. Suicide knowledge and intention to intervene: college students. J Am Coll Heal. (2021) 71:182–9. 10.1080/07448481.2021.1885413 33759712

