# Supplementary material for: Suicide stigma and suicide literacy among Bangladeshi young adults: a cross-sectional study
Source: Front Psychiatry. 2023 May 12;14:1160955. doi: 10.3389/fpsyt.2023.1160955 (PMC10213423; doi:10.3389/fpsyt.2023.1160955)
Supplement: Supplementary file 1 [file Table_1.DOCX]

Supplementary Material

Suicide stigma and suicide literacy among Bangladeshi young adults: A cross-sectional study

Ishrat Jahan, Azaz Bin Sharif ^*^, A.B.M. Nahid Hasan

*** Correspondence:** Azaz Bin Sharif: azaz.sharif@northsouth.edu

**1** Supplementary Tables

Table S1. Items of the stigma of suicide scale (SOSS-SF) with responses of the participants who agree or strongly agree (N=616) put it in supplementary

| ***Subscale (Mean ± SDᵈ)*** | ***Item*** | ***Agree/Strongly agree (%)*** | ***Mean ± SDᵈ*** |
| --- | --- | --- | --- |
| Stigmatization (25.15 ± 6.16) | Immoral | 36.4 | 3.00 ± 1.25 |
|  | Irresponsible | 47.2 | 3.20 ± 1.20 |
|  | Pathetic | 53.4 | 3.35 ± 1.14 |
|  | Stupid | 55.3 | 3.40 ± 1.22 |
|  | Cowardly | 38.3 | 2.96 ± 1.21 |
|  | Vengeful | 19.8 | 2.66 ± 1.00 |
|  | Shallow | 65.8 | 3.62 ± 1.23 |
|  | Embarrassment | 36.1 | 2.96 ± 1.15 |
| Isolation (14.48 ± 2.91) | Lonely | 83.6 | 4.01 ± 0.91 |
|  | Isolated | 73.7 | 3.80 ± 0.93 |
|  | Disconnected | 46.3 | 3.31 ± 0.99 |
|  | Lost | 49.5 | 3.35 ± 1.05 |
| Glorification (9.04 ± 2.69) | Noble | 4.5 | 1.99 ± 0.93 |
|  | Strong | 7.8 | 1.96 ± 0.90 |
|  | Dedicated | 9.9 | 2.44 ± 0.93 |
|  | Brave | 28.1 | 2.64 ± 1.19 |
| **Note: *ᵈ* Standard Deviation** | | | |

Table S2. Correlation between literacy of suicide scale (LOSS-SF) and three subscales of stigma of suicide scale (SOSS-SF) put it in supplementary

|  | **SOSSᶠ** | | | | |
| --- | --- | --- | --- | --- | --- |
|  |  | **LOSSᵉ** | **Stigma** | **Isolation** | **Glorification** |
|  | **1.LOSSᵉ** |  |  |  |  |
| **SOSS** | **2.Stigma** | -0.113** |  |  |  |
|  | **3.Isolation** | 0.014 | 0.270*** |  |  |
|  | **4.Glorification** | -0.053 | -0.180*** | -0.136** | - |
|  | **Note. *p<0.05; **p<0.01; ***p<0.001**  **ᵉLiteracy of Suicide Scale**  **ᶠStigma of Suicide Scale** | | | | |
